# International mobility, sexual behaviour and HIV-related characteristics of men who have sex with men residing in Belgium

**DOI:** 10.1186/1471-2458-13-968

**Published:** 2013-10-18

**Authors:** Wim Vanden Berghe, Christiana Nöstlinger, Harm Hospers, Marie Laga

**Affiliations:** 1 Department of Public Health, Institute of Tropical Medicine HIV/AIDS Center (IHAC), Antwerp, Belgium; 2 University College Maastricht, Maastricht University, Maastricht, The Netherlands

**Keywords:** HIV infection, Men who have sex with Men (MSM), Travel

## Abstract

**Background:**

European men who have sex with men (MSM) continue to be disproportionally affected by the human immunodeficiency virus (HIV). Several factors are contributing to the rates of new HIV infections among MSM. The aim of this study was to investigate the potential role of travel behaviour and sexual mobility in the spread of HIV and sexually transmitted infections (STI) among European MSM.

**Methods:**

Belgian data from the first pan-European MSM internet survey EMIS was used (n=3860) to explore individual and contextual determinants of sexual behaviour among MSM, who resided in Belgium at the time of data collection and who reported having had sexual contact abroad in the last 12 months. Descriptive and bivariate analyses were performed. Odds ratios and 95% confidence intervals were calculated by means of logistic regression.

**Results:**

MSM who practiced unprotected anal intercourse UAI during their last sexual encounter abroad were less likely to be living in a large city (OR:0.62, 95% CI:0.45-0,86, p<0.01) and more likely to be HIV positive (OR:6.20, 95% CI:4.23-9.06, p<0.001) ), to have tested HIV positive in the last 12 months (OR:3.07, 95% CI:1.07-8.80, p<0.05), to have been diagnosed with any STI in the last 12 months (OR:2.55; 95% CI:1.77-3.67, p<0.05), to have used party drugs (OR:2.22, 95% CI:1.59-3.09, p<0.001), poppers (OR:1.52, 95% CI:1.07-2.14, p<0.001) and erection enhancing substances (OR:2.23, 95% CI:1.61-3.09, p<0.001) compared to MSM who did not have UAI with their last sexual partner abroad. Men having had UAI in the last 12 months were more likely to have done so in a neighbouring country of Belgium (OR:1.66, 95% CI:1.21-2.29, p<0.001). Different sexual behavioural patterns related to condom use and drug use were identified according to HIV test status among travelling men.

**Conclusions:**

The results of this study provide evidence for the role of international mobility and sexual behavior while travelling, in the spread of HIV and STI among MSM in Europe. Further, the findings underline the need for development of European cross-border HIV and STI interventions with coherent messages and prevention policies for MSM.

## Background

The human immunodeficiency virus (HIV) continues to disproportionally affect men who have sex with men (MSM), an epidemic that remains a challenge for European public health. In most Western European countries, including Belgium, the number of yearly diagnoses of new HIV infections in MSM remains high [1,2]. Several factors are contributing to the rates of new HIV infections among MSM, including an increase in sexual risk behaviour, combined with a high prevalence of HIV infection in some networks of MSM [3-5]. It has also been suggested that travel behaviour and sexual mobility could potentially be a factor in the spread of HIV and sexually transmitted infections (STI) among European MSM [1,4]. Travelling has been considered an important component of gay lifestyle and identity, as gay men travel to destinations where they can socialize with peers and in some cases avoid the social constraints and intolerance of their home environments [6]. For some gay men, social and sexual networking are highly intertwined and sexual identity is a central factor for some gay men in deciding on the travel destination [7]. Furthermore, globalization and European integration have resulted in shifts in travel decision making, among other reasons because of cheaper flights, which made gay men and other MSM even more internationally mobile than before. These processes have not developed to the same extent across Europe, and some countries have a different general tourism profile than others [8,9]. At European level, most Western European countries have been at the international forefront in implementing legal equality and anti-discrimination laws for lesbians, gays, bisexuals and transgenders (LGBT). In this changing context, some bigger cities such as Barcelona (ES), Berlin (DE) or Paris (FR), but also Antwerp (BE) have invested in- and attracted more LGBT tourism by means of different social marketing strategies. These cities offer a diverse mixture of cultural and festive experiences directed at sexual minorities and especially MSM, mirroring the diversity of gay subculture. These efforts seem to influence the choice of travel destination for MSM. For instance, results from the recent European MSM Internet study (EMIS) showed that Spain, Germany and France were the countries to which the majority of MSM traveled [10,11]. The factors above may explain the higher probability of finding more gay men and in a broader sense, MSM, in a given travel destination.

There is a need to better understand the dynamics that are at the basis of the European HIV/STI epidemics among MSM, particularly how sexual networks across countries may contribute to the current HIV epidemic among European MSM. Different United States-based studies showed that, in the context of recreational travel, MSM reported specific patterns of unprotected anal intercourse (UAI) and related substance use [12,13]. However, limited attention has been paid thus far to investigate sexual behaviour of European gay men while travelling. The present study aims at filling this gap by analysing HIV-related individual- and contextual characteristics of MSM residing in Belgium travelling abroad. Belgium functions here as a case study for sexual behaviour among mobile MSM in the European context. We use data from the first pan-European MSM internet survey EMIS to explore individual and contextual determinants of sexual behaviour among MSM who were residing in Belgium at the time of data collection and who reported having had sexual contact abroad in the preceding 12 months.

## Methods

### Dataset

The European MSM Internet survey recruited participants from June up to and including August 2010. MSM from 38 different European countries, including the 27 EU Member States, completed an online questionnaire on sexual health, which was presented in 25 languages. An offline- and online recruitment campaign, with the support of many LGBT organizations across all countries, preceded the data collection to motivate respondents to participate. The use and support of gay social online networks *Gayromeo*, *Manhunt*, *Qruiser*, *Qguys* and *Gaydar* were instrumental in reaching the target population. In total 174209 MSM participated in the EMIS study. Respondents completed the questionnaire in an average time of 20 minutes. More information and on the EMIS project and its methodology and descriptive results can be found on http://www.EMIS-project.eu, in a number of publications [14-16] and in the EMIS final report [11]. The EMIS study was approved by the Research Ethics Committee of the University of Portsmouth, UK (REC application number 08/09:21).

### Measures

#### ***Background characteristics***

Respondents reported on their age, education, sexual identity, level of urbanization (i.e. living in a city with more than 500.000 inhabitants), occupation, nationality, EMIS expat- (living in one of the 38 EMIS countries but not in the country of birth) and relationship status.

#### ***Sexual and HIV/STI related behaviour, drug use and health aspects in the last 12 months***

With regards to sexual practices, we assessed whether participants had anal sex and if so, whether condoms were used with casual and steady partners during 12 months before the EMIS study. We refer to anal sex without the use of condoms as unprotected anal intercourse or UAI. We assessed perceived seroconcordant UAI with a casual partner in the last 12 months. The participants also reported on HIV and STI testing behaviour (Syphilis, Gonorrhea, Chlamydia, anal/genital warts, anal/genital herpes, Hepatitis B and C) and diagnoses during the last 12 months, the current HIV serostatus, the number of non-steady sex partners. We assessed recreational drugs typically associated with sex and parties referring to them in this paper as party drugs (XTC, amphetamine, crystal, mephedrone, GHB, ketamine, cocaine), poppers, and erectile enhancing medication use. HIV positive participants reported whether they were taking antiretroviral treatment (ART) and their HIV viral load during their last check-up.

#### ***Sexual contact abroad***

Respondents reported whether they have had any sexual contact with casual partners abroad in the last 12 months. Subsequently, more detailed questions were asked related to the last sexual contact abroad: country in which it took place; how and where the sexual partner was met, whether they engaged in anal sex and if so, whether condoms were used.

### Statistics

Data analysis was performed using IBM SPSS statistics version 20. To assess differences in proportions and means, according to having had sex abroad, Mann–Whitney U-tests were performed for continuous variables, and chi squares were calculated for categorical variables. Univariate and multivariate logistic regression was used to calculate odds ratios and 95% confidence intervals for the different background, risk and contextual factors comparing MSM on the basis of having had sex abroad in the last 12 months and having performed UAI during the last sexual encounter abroad. Adding variables to the model for the multivariate analysis was based on significance of the univariate analyses. Differences in proportions and means between three different groups (i.e. HIV positive, HIV negative, and untested men) on the basis of self-reported HIV-status were tested for statistical significance using analysis of variance (ANOVA) and Tukey’s post-hoc test.

## Results

### Description of the total sample of MSM residing in Belgium

The total EMIS sample of MSM residing in Belgium comprised 3982 men. We excluded 84 participants who said they never had sex with another man and 38 men who did not answer the questions on sexual contact abroad. The total number of eligible men for analysis was 3860 (Table 1). Mean age was 35.2 (SD:11.4) years old. Of Belgian resident MSM, 64.6% (n:2493) were higher educated, 5.2% (n:200) were unemployed and 45.5% (n:1758) were in a steady relationship. A total of 955 (24.7%) men were expatriates and 43.3% (n:1674) resided in a large city. Concerning sexual identity, 85.4% (n:3298) reported to identify as homosexual, 9% (n:348) as bisexual. During the last 12 months, 85% (n:3281) of respondents reported having had anal sex, 39.2% (n:1514) had UAI with a steady partner and 25.7% (n:993) had UAI with casual partner(s). Overall, 43.7% (n:1688) of MSM had an HIV test in the last 12 months and 10.4% (n:332) reported being HIV positive. A total of 1562 (40.5%) respondents had been tested for STI in the last 12 months and the most frequently reported STI were Syphilis (7.7%, n:121), Gonorrhea (8.8%, n:137) and Chlamydia (8.3%, n:130). For all respondents, substance use in the last 12 months included consuming party drugs (17.7%, n:683), poppers (38.9%, n:1500) and erectile enhancers (19.5%, n:751). Of central interest to this study is that 39.1% (n:1510) of Belgian resident MSM reported having had sexual contact abroad with a casual partner in the last 12 months. We will refer to this group of MSM as “sexually mobile men”.

**Table 1:** Hiv related individual and contextual characteristics of MSM residing in Belgium according to having had sexual contact abroad in the last 12 months

	**MSM residing in Belgium - Sexual contact abroad?**						Test statistic*	P value**
	**Total (N:3860)**	**%**	**Yes (N:1510)**	**%**	**No (N:2350)**	**%**		
**Age (mean(SD))**	3860	35,2(11,4)	1510	37,2 (10,33)	2350	34,2(12,1)	62,408	<0,001
**Higher education**	2493	64,6	1077	71,3	1416	60,3	82,306	<0,001
**Unemployment rate**	200	5,2	70	4,6	130	5,5	10,426	0,001
**Steady relationship**	1758	45,5	669	44,3	1089	46,3	0,04	0,433
**Expatriate**	955	24,7	581	38,5	374	15,9	251,325	<0,001
**Living in a large city (>500.000 inh.)**	1674	43,4	828	54,8	846	36	140,557	<0,001
**Sexual identity - Homosexual**	3298	85,4	1344	89	1954	83,1	26,129	<0,001
**Sexual identity - Bisexual**	348	9	92	6,1	256	10,9	25,77	<0,001
**Anal sex last 12 m**	3281	85	1406	93,1	1860	79,1	145,244	<0,001
**UAI Steady partner last 12 m**	1514	39,2	5703	37,7	940	40	2,265	0,072
**UAI Casual partner last 12 m**	993	25,7	551	36,5	437	18,6	32,812	<0,001
**Nr of casual partners last 12 m (median category)**		4		11 to 20		2	1227,601	<0,001
**Hiv test last 12 m**	1688	43,7	718	47,5	970	41,3	48,445	<0,001
**Hiv status **- **positive (among tested)**	332	10,4	210	15,2	122	6,8	58,433	<0,001
**STI test last 12 m**	1562	40,5	805	53,3	757	32,2	96,565	<0,001
**STI history last 12 m (among tested for any STI)**								
Syphilis	121	7,7	75	9,3	46	6,1	27,299	<0,001
Gonorrhoea	137	8,8	92	11,4	45	5,9	46,995	<0,001
Chlamydia	130	8,3	95	11,8	35	4,6	65,283	<0,001
Anal/genital warts	98	6,3	54	6,7	44	5,8	10,92	0,001
Anal/genital herpes	42	2,7	22	2,7	20	2,6	3,135	0,055
Hepatitis C 12 m	10	0,6	6	0,7	4	0,5	1,839	0,152
Hepatitis C current	27	1,7	16	2,0	11	1,5	4,638	0,027
Hepatitis B (ever)	289	18,5	153	19,0	136	18,0	25,05	0,001
**Drug use last 12 m**								
Party drugs	683	17,7	407	26,9	276	11,8	146,085	0,001
Poppers	1500	38,9	850	56,3	650	27,7	179,881	0,001
Erectile enhancers	751	19,5	465	30,8	286	12,2	202,213	0,001
**Anal sex during last sexual contact abroad**			859	56,8				
**UAI during last sexual contact abroad**			219	14,5				
**Country of last sexual contact abroad**								
The Netherlands			137	9,1				
Germany			161	10,6				
France			272	18,1				
UK			74	4,9				
Spain			237	15,7				

*Test statistics: U value for continuous variables. χ2 for categorical variables.

**p values: Mann–Whitney U test for continuous variables and χ2 for categorical variables.

### MSM residing in Belgium

#### ***Differences according to having had sexual contact abroad in the last 12 months***

Table 1 displays the bivariate analysis of the different background, sexual risk and contextual characteristics of MSM residing in Belgium according to having had sex abroad with a casual partner in the last 12 months. Significant differences are reported between sexually mobile MSM, compared to those men residing in Belgium, who did not have sex abroad. Sexually mobile men were on average significantly older (37.2 vs. 34.2 years old, p<0.001), had a higher education (71.3% vs. 60.3%, p<0.001), were more often living in Belgium as an expatriate (38.5% vs.15.9%, p<0.001), and resided more often in a large city (54.8% vs. 36%, p<0.001), compared to men who did not have sex abroad. Also, sexually mobile men identified more often as homosexual or gay (89.0% vs. 83.1%, p<0.001) and less often as bisexual (6.1% vs. 10.9%, p<0.001). Further, HIV testing (47.5% vs. 41.3%, p<0.001) and STI testing (53.3% vs. 32.2%, p<0.001) in the last 12 months was more often reported and a higher percentage of sexually mobile men were HIV positive (15.2% vs. 6.8%, p<0.001). Higher numbers of diagnoses of Syphilis (9.3% vs. 6.1%, p<0.001), Gonorrhea (11.4% vs. 5.9%, p<0.001), Chlamydia (11.8% vs. 4.6%, p<0.001)), anal/genital warts (6.7% vs. 5.8%, p<0.01), current infection of hepatitis C (2% vs. 1.5%, p<0.05) during the last 12 months and lifetime infection of hepatitis B (19% vs. 18%, p<0.001) were reported by sexually mobile men compared to men who did not have sex abroad. Sexually mobile men more often had anal sex (93.1% vs. 79.1%, p<0.001) and more unprotected anal sex with casual partner(s) (36.5% vs. 18.6%, p<0.001) in the last 12 months. They also had a higher number of non-steady sex partners in the last 12 months (11 to 20 vs. 2 partners, p<0.001), used more party drugs (26.9% vs. 11.8, p<0.001), poppers (56.3% vs. 27.7%, p<0.001) and erection enhancing substances (30.8% vs. 12.2%, p<0.001) in the last 12 months compared to Belgian resident MSM who did not have any sexual contact abroad. Countries, where a majority of sexually mobile men had sexual contacts during the last 12 months included France (18.1%), Spain (15.7%), Germany (10.6%) and The Netherlands (9.1%).

#### ***Determinants of UAI during the last sexual encounter abroad among sexually mobile men***

Table 2 shows the results of univariate and multivariate logistic analyses with UAI during the last sexual encounter abroad as the dependent variable. Sexually mobile men, who practiced UAI during their last sexual encounter abroad were less likely to be living in a large city (OR:0.62, 95% CI:0.45-0,86, p<0.01) and more likely to be HIV positive (OR:6.20, 95% CI:4.23-9.06, p<0.001)), to have been diagnosed HIV positive in the last 12 months (OR:3.07, 95% CI:1.07-8.80, p<0.05), to have been diagnosed with any STI in the last 12 months (OR:2.55; 95% CI:1.77-3.67, p<0.05), to have used party drugs (OR:2.22, 95% CI:1.59-3.09, p<0.001), poppers (OR:1.52, 95% CI:1.07-2.14, p<0.001) and erection enhancing substances (OR:2.23, 95% CI:1.61-3.09, p<0.001), compared to MSM who did not have UAI with their last sexual partner abroad. Men having had UAI in the last 12 months were more likely to have done so in a neighbouring country of Belgium (OR:1.66, 95% CI:1.21-2.29, p<0.001).

**Table 2:** Univariate and multivariate logistic regression with unprotected anal intercourse during the last sexual encounter of MSM residing in Belgium as the dependent variable

	**UAI during last sexual encounter abroad**							
	**Univariate**				**Multivariate**			
	**P**	**OR**	**95% CI**		**P**	**OR**	**95% CI**	
**Age >= 30 years old**	0,940	1,01	0,71	1,44				
**Higher education**	0,153	0,77	0,54	1,10				
**Steady relationship**	0,208	1,23	0,89	1,69				
**Expatriate**	0,480	0,89	0,64	1,24				
**Living in a large city (>500000 inh.)**	0,004	0,62	0,45	0,86	0,041	0,67	0,46	0,98
**Diagnosed HIV positive last 12 m**	0,036	3,07	1,07	8,81				
**Diagnosed with any STI last 12 m**	<0,001	2,55	1,77	3,68	0,019	1,76	1,10	2,83
**Hiv status – positive**	0,001	6,20	4,24	9,07	0,009	3,88	1,41	10,67
**Number of non**- **steady partners last 12 m (1 to 5)**								
6 to 20	0,345	0,81	0,53	1,25				
20 +	0,306	1,24	0,82	1,87				
**Party drugs**	<0,001	2,22	1,60	3,10	0,912	1,03	0,63	1,69
**Poppers**	<0,001	1,52	1,08	2,14	0,632	0,90	0,60	1,36
**Erectile enhancers**	<0,001	2,23	1,61	3,09	0,757	1,07	0,69	1,68
**Sexual contact in neighbouring country**	<0,001	1,67	1,21	2,30	0,711	1,08	0,73	1,59
**Meeting place for last sexual contact abroad**								
Website	0,321	0,85	0,62	1,17				
Gay bar/disco/nightclub	0,923	1,02	0,67	1,55				
Sex club/darkroom/gay sauna	0,701	1,07	0,77	1,47				

Multivariate analysis with MSM having had UAI abroad as the dependent variable showed that, when controlling for variables significant in the univariate models, the odds for having had UAI during the last sexual encounter abroad were 3,88 times higher for HIV positive men (95% CI:1.14-10.67, p<0.01), 1.76 higher for men, diagnosed with any STI in the last 12 months (95% CI:1.1-2.83, p<0.05) and 0.67 lower for men, residing in a large city (95% CI:0.46-0.98, p<0.05).

#### ***Sexual behaviour, drug use and STI history in the last 12 months according to HIV test status among sexually mobile men***

Table 3 shows characteristics of the last sexual contact abroad according to participants’ HIV test status. Of the HIV positive men, 62% were on ART and 57% had an undetectable viral load. HIV positive men reported significantly more often to have had unprotected sex with a casual partner (73.9 vs. 34% vs. 27.1%, p<0.001), practiced more seroconcordant UAI with a casual partner in the last 12 months (52.8% vs. 15.8% vs. 12.9%, p<0.001) and had a higher number of casual sex partners during the last 12 months than HIV negative and untested men (more than 50 vs. 11 to 20 partners, p<0.001). Concerning STI history, HIV positive men reported significantly more Syphilis (43.9% vs. 18.8% vs. 8.3%, p<0.001) and Chlamydia (19.7% vs. 4.3% vs. 3.4%) during the last 12 months and more current infections of Hepatitis C (14.6% vs. 1.9% vs. 8.3%), p<0.001); than HIV negative and untested men. Party drugs (62.7% vs. 22.2% vs. 12.9%, p<0.001), poppers (62.7 vs. 26.6% vs. 13.8%) and erection enhancing substances (79.8% vs. 54.2% vs. 33.6%) were used significantly more often by HIV positive men than by HIV negative and untested men.

**Table 3:** Background and HIV-related characteristics according to HIV test status among mobile MSM residing in Belgium*

	**Hiv positive (P)**		**Hiv negative (N)**		**Untested (U)**		**Anova - P value**	**Tukey's test**
	**N**	**%**	**N**	**%**	**N**	**%**		
**Age (mean (SD))**	193	40(9,47)	1117	36,99(10,5)	116	34,38(12,59)	<0,001	P≠N≠U
**On antiretroviral therapy**	129	62						
**Viral load (last checkx-up) undetectable**	114	57						
**Anal sex last 12 m**	186	96,4	1041	92,5	106	85,5	0,038	P≠U
**UAI Steady partner last 12 m**	94	48,7	425	61,2	31	54	0,002	P≠N,U
**UAI Casual partner last 12 m**	137	71	347	34	28	27,1	<0,001	P≠N,U
**Perceived seroconcordant UAI with a casual partner last 12 m**	102	52,8	172	15,4	15	12,9	<0,001	P≠N,U
**Nr of casual partners last 12 m (median category)**		More than 50		11 to 20		11 to 20	<0,001	P≠N≠U
**STI test last 12 m**	82	42,5	160	14,5	12	10,3	<0,001	P≠N,U
**STI history last 12 m (among tested for any STI)**								
Syphilis	36	43,9	30	18,8	1	8,3	<0,001	P≠N,U
Gonorrhoea	29	35,4	53	33,1	5	41,7	<0,001	U≠P,N
Chlamydia	38	46,3	48	30,0	4	33,3	<0,001	P≠N,U
Anal/genital warts	14	17,1	32	20,0	5	41,7		P≠N
Anal/genital herpes	3	3,7	16	10,0	0	0,0	0,865	
Hepatitis C 12 m	3	3,7	1	0,6	0	0,0	<0,001	P≠N,U
Hepatitis C current	12	14,6	3	1,9	1	8,3	<0,001	P≠N,U
Hepatitis B (ever)	42	51,2	89	55,6	5	41,7	<0,001	P,N≠U
**Drug use last 12 m**								
Party drugs	121	62,7	248	22,2	15	12,9	<0,001	P≠N,U
Poppers	121	62,7	297	26,6	16	13,8	<0,001	P≠N≠U
Viagra	154	79,8	605	54,2	39	33,6	<0,001	P≠N≠U
**Anal sex during last sexual contact abroad**	160	82,9	627	56,1	69	59,5	<0,001	P≠N,U
**UAI during last sexual contact abroad**	91	47,2	115	10,3	10	8,6	<0,001	P≠N,U
**Meeting place for last sexual contact abroad**								
Gay bar/disco/nightclub	34	17,6	220	19,7	14	12,1	0,147	
Sex club/Darkroom	38	19,7	107	9,6	4	3,4	<0,001	P≠N,U
A gay sauna	22	11,4	176	15,8	17	14,7	0,247	
Public cruising location	20	10,4	116	10,4	13	11,2	0,985	
Website	71	36,8	372	33,3	51	44,0	0,177	

*Total n for sexual contact abroad among MSM residing in Belgium = 1510 (may differ due to missing values on HIV test status).

#### ***Characteristics of the last sexual encounter abroad according to HIV test status among sexually mobile men***

HIV-positive men reported higher numbers of anal sex (82.9% vs. 56.1%; 59.5%, p<0.001) and also more occasions of unprotected anal sex than HIV negative and untested men (47.2% vs. 10.3%; 8.6%, p<0.001). HIV positive men also met their last sex partner abroad significantly more often in sex clubs and darkrooms (19.7% vs. 9.6%; 3.4%, p<0.001) than their HIV-negative or untested counterparts.

## Discussion

The present study used data from the European Internet MSM Survey (EMIS) to investigate sexual behaviour of MSM who were sexually active when travelling abroad in the last 12 months prior to the survey. Our results for the case of Belgium show that HIV and STI are highly prevalent among travelling MSM, and that they adopt more sexual risk behaviours than MSM who were not sexually active abroad. This evidence supports cross-border development of prevention activities for HIV and STI among MSM.

MSM residing in Belgium who had sexual contact abroad were more likely to be expatriates and to be living in a bigger city. This finding can probably be explained by the substantial population of expatriates living in Brussels and working at its many European and international institutions. Belgian resident MSM having had sex abroad were also better educated and on average older than their counterparts who did not report any sexual activity abroad. Being able to travel implies disposable income to do so. Similar results were found in a study on gay resorts [17]. This study also demonstrates that sexual identity does play a role in travel behaviour as was suggested in other studies [6,10,18]. Significantly more sexually mobile MSM identified as homosexual or gay and less often as bisexual. The majority of MSM residing in Belgium who had sex abroad mostly did so in Belgium’s neighboring countries: Germany, France, The Netherlands and the United Kingdom, but also in Spain. Local evidence corroborates this cross border mobility. Of the 642 participants in a recent venue-based HIV prevalence study among MSM in Antwerp, Belgium, 1 in 5 men were of non-Belgian nationality, mostly Dutch [4]. These findings are in line with the mobility dynamics of European MSM. According to the EMIS final report, Spain and Germany are the most frequented countries for having sex abroad [11].

Our findings showed that the proportion of HIV-positive men was higher among sexually mobile men. This may be due to a higher number of casual sex partners and more UAI with these casual sex partners, although it cannot be established to what extent seroconversion affected the sexual behaviour of the now positive men. Sexually mobile men were also tested for HIV and STI more frequently during the last 12 months. However, mobile positive men reported higher rates of STI and substance use, and lower rates of condom use during anal sex while seroconcordant UAI with a casual partner in the last 12 months was higher compared to negative and untested MSM. This is in line with other research pointing to the fact that MSM and specifically some networks of HIV positive MSM are highly mobile and apply additional risk reduction strategies next to condom use based on their own and their partner’s HIV-status, HIV treatment and viral load status [19,20]. However, these strategies do not seem to protect well against other STIs. Over the last years networks of HIV positive men have been confronted with several co-occurring epidemics of for instance Lymphogranuloma Venereum (LGV) [21] and more recently Hepatitis C [1,22-24]. Our study corroborated a link between the use of specific types of substance use, a higher number of non-steady partners and visiting patterns of specific venues where sexual contact is facilitated, which has also been established in other research [4,20,25-27]. Over one third of sexually mobile men searched for a sex partner through the Internet for their last sexual encounter abroad, confirming findings from other studies [12]. However, this study did not find an association between UAI abroad and searching for sex partners through the Internet before or during travel. An important finding in our study is that Belgian resident men, who tested HIV positive in the last 12 months, were more likely to report UAI abroad. This finding suggests that international sexual mobility may well have contributed to recent infections with HIV among European MSM. However, the data does not allow to pinpoint the exact moment of seroconversion in the last 12 months and an HIV positive diagnosis during that period may well have influenced the individual’s sexual behaviour and eventually the likelihood of performing UAI.

It has to be stressed that these results cannot be generalized to all MSM, HIV negative or positive, because a majority of the study participants were recruited through specific Internet sites aiming at social and sexual networking. Being more active on these Internet sites could be an indicator of heightened sexual activity. However, the strengths of this study are its large sample size and anonymity of data collection through the Internet, which may also contribute to the validity of sensitive data [14,28]. Another limitation is that the EMIS survey did not investigate travel motivations or other more in-depth factors connected to travel behaviour.

## Conclusions

The results of this EMIS study provide evidence for the role of international mobility and sexual behaviour regarding the spread of HIV and STI among MSM in Europe, while travelling abroad. As the epidemiology of HIV infection among Belgian MSM mirrors developments in its neighbouring countries, the findings underline the need for development of cross-border HIV and STI interventions with coherent messages and prevention policies for MSM. Systematic cross-border cooperation and exchange of epidemiological and behavioural data for the development of prevention activities for MSM at a regional European level should inform future prevention effort for the target population. Further research should explore in more detail the purpose of travelling and context-related characteristics that lead MSM to be sexually active while being abroad. This could inform the development of targeted prevention campaigns supporting sexually mobile MSM to reduce high risk behaviour. The presented evidence also underscores the need for multiple-behaviour interventions, targeting not only sexual behaviour but also substance use. In this context, multiple-behaviour interventions could have a greater impact on public health than single-behaviour interventions [29,30]. Further research is needed on development of effective multiple-behaviour interventions for this target group, which also must be tailored to the needs of specific subgroups such as HIV positive men [20,31-33] and HIV negative men, who are at greater risk for infections with HIV/STI [34].

## Competing interests

The authors declare that they have no competing interest.

## Authors’ contributions

The work presented here was carried out in collaboration between all authors. WV and HH defined the research theme. WV analyzed the data. WV, CN, HH and ML all interpreted the results and co-wrote the paper. All authors have contributed to, seen and approved the manuscript.

## Pre-publication history

The pre-publication history for this paper can be accessed here:

http://www.biomedcentral.com/1471-2458/13/968/prepub
